# PR‐DUB safeguards Polycomb repression through H2AK119ub1 restriction

**DOI:** 10.1111/cpr.13457

**Published:** 2023-03-23

**Authors:** Rui Li, Dandan Huang, Yingying Zhao, Ye Yuan, Xiaoyu Sun, Zhongye Dai, Dawei Huo, Xiaozhi Liu, Kristian Helin, Mulin Jun Li, Xudong Wu

**Affiliations:** ^1^ State Key Laboratory of Experimental Hematology, The Province and Ministry Co‐sponsored Collaborative Innovation Center for Medical Epigenetics, Key Laboratory of Immune Microenvironment and Disease (Ministry of Education), Department of Cell Biology, School of Basic Medical Sciences Tianjin Medical University Tianjin 300070 China; ^2^ Wuxi School of Medicine Jiangnan University Wuxi 214000 China; ^3^ Pediatric Center, Tianjin Key Laboratory of Epigenetics for Organ Development of Premature Infants The Fifth Central Hospital of Tianjin Tianjin 300450 China; ^4^ Biotech Research and Innovation Centre University of Copenhagen Copenhagen Denmark; ^5^ The Institute of Cancer Research (ICR) London UK; ^6^ Department of Bioinformatics, School of Basic Medical Sciences Tianjin Medical University Tianjin 300070 China; ^7^ Department of Epidemiology and Biostatistics, National Clinical Research Center for Cancer, Tianjin Medical University Cancer Institute and Hospital Tianjin Medical University Tianjin 300070 China; ^8^ Department of Orthopedics Tianjin Medical University General Hospital Tianjin 300052 China

## Abstract

Polycomb group (PcG) proteins are critical chromatin regulators for cell fate control. The mono‐ubiquitylation on histone H2AK119 (H2AK119ub1) is one of the well‐recognized mechanisms for Polycomb repressive complex 1 (PRC1)‐mediated transcription repression. Unexpectedly, the specific H2AK119 deubiquitylation complex composed by additional sex comb‐like proteins and BAP1 has also been genetically characterized as Polycomb repressive deubiquitnase (PR‐DUB) for unclear reasons. However, it remains a mystery whether and how PR‐DUB deficiency affects chromatin states and cell fates through impaired PcG silencing. Here through a careful epigenomic analysis, we demonstrate that a bulk of H2AK119ub1 is diffusely distributed away from promoter regions and their enrichment is positively correlated with PRC1 occupancy. Upon deletion of *Asxl2* in mouse embryonic stem cells (ESCs), a pervasive gain of H2AK119ub1 is coincident with increased PRC1 sampling at chromatin. Accordingly, PRC1 is significantly lost from a subset of highly occupied promoters, leading to impaired silencing of associated genes before and after lineage differentiation of *Asxl2*‐null ESCs. Therefore, our study highlights the importance of genome‐wide H2AK119ub1 restriction by PR‐DUB in safeguarding robust PRC1 deposition and its roles in developmental regulation.

## Graphical abstract

A model for understanding why PR‐DUB acts as a PRC despite of an opposite biochemical activity against PRC1. PR‐DUB prevents H2AK119ub1 at the genome wide. Upon delimitation from PR‐DUB, increased H2AK119ub1 activity at non‐promoters is associated with favourable yet unstable RING1B occupancy. Accordingly, PRC1 is diluted from highly occupied promoters, leading to impaired PRC silencing.

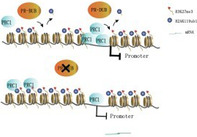

## 1 INTRODUCTION

The precise control of cellular fidelity and plasticity is paramount for the development of multicellular organisms. Epigenetic regulators are central players for the inheritance and flexibility of transcriptional programmes. Among them, Polycomb group (PcG) proteins are evolutionarily conserved chromatin repressors and critical for the maintenance of cell memory. The deregulation of PcG functions has been shown to cause aberrant differentiation and development and human diseases including cancers.^1^, ^2^, ^3^

Biochemically, PcG proteins usually form multi‐subunit transcription repressive complexes, known as Polycomb repressive complexes 1 and 2 (PRC1 and PRC2). PRCs are preferentially deposited at inactive promoters to maintain target gene silencing through either histone modifications or chromatin compaction.^4^, ^5^ Briefly, PRC2 is mainly composed of EED, SUZ12 and EZH1/2 and catalyses the methylation of lysine 27 at histone H3 (H3K27me). Among PRC1s, canonical (cPRC1) contributes to chromatin compaction while non‐canonical or variant PRC1 (ncPRC1 or vPRC1) is responsible for the deposition of mono‐ubiquitylation on H2AK119 (H2AK119ub1).^5^, ^6^ In ncPRC1s, RING1B is the core E3 ligase while RYBP facilitates the catalytic activity and recognizes H2AK119ub1 for its propagation.^7^

Ever since H2AK119ub1 was found to be catalysed by PRC1‐like complex or ncPRC1,^8^ this mark has been tightly associated with transcription repression. Recent evidences have supported a central role of H2AK119ub1 in the maintenance of transcription repression at least in mouse embryonic stem cells (mESCs).^9^, ^10^, ^11^ We have previously identified and characterized specific mammalian H2AK119 deubiquitylation complex^12^ which was earlier known as Polycomb repressive deubiquitinase (PR‐DUB) in *Drosophila*.^13^ The complex is mainly composed of BRCA1 associated protein 1 (BAP1) and additional sex comb (Asx)‐like proteins (ASXLs), in addition to a set of accessory proteins, such as FOXK1/2, HCF‐1 and the O‐GlcNAc transferase OGT. BAP1 is a ubiquitin hydrolase to specifically remove ubiquitin from H2AK119 while each of the ASXLs (ASXL1, ASXL2, and ASXL3) is indispensable for the H2AK119 deubiquitylation activity.^12^, ^14^, ^15^, ^16^, ^17^ However counterintuitively, the loss of this complex was found to mimic the phenotypes of PcG mutants and PR‐DUB was therefore genetically identified as a transcription repressor in *Drosophila*.^13^ In line with this, ASXL or BAP1 depletion in mammalian cells has been shown to cause depression of PcG target genes.^18^, ^19^, ^20^ And a few of previous studies have even demonstrated that ASXL1/2 or BAP1 is required for the maintenance of global or local H3K27me3 levels.^18^, ^21^, ^22^, ^23^ Accordingly, a model has been proposed that PR‐DUB physically interacts with PRC2,^18^ though it does not seem a general mechanism.^12^, ^24^, ^25^ Recently, two independent studies have shown that BAP1 loss from ESCs causes PRC1/2 dissociation from a subset of target promoters and thereby affects their roles in gene silencing.^26^, ^27^ A more intriguing finding is that H2AK119ub1 directly resists chromatin compaction, which is actually fostered by cPRC1.^28^ However, it remains uncertain how these dynamic changes of different PRCs are correlated with H2AK119ub1 alterations at the genome wide.

Here we generate PR‐DUB or PRC1 inactivation mESC models to re‐examine the roles of H2AK119ub1. Interestingly, we find that H2AK119ub1 is enriched far more than at promoters and its levels at non‐promoter regions are associated with low levels/frequencies of RING1B binding. The pervasive gain of non‐promoter H2AK119ub1 in *Asxl2*‐null cells leads to widespread re‐localization of PRC1 from dominantly repressed promoters to acquired weak occupancy sites and thereby undermines gene silencing. Taking advantage of lineage differentiation models, we show that ASXL2 loss causes aberrant lineage specification, a typical phenotype of *PcG* mutants. These findings provide a novel insight into how PR‐DUB restricts H2AK119ub1 and prevents PRC1 roaming so as to safeguard PcG functions.

## 2 MATERIALS AND METHODS

### 2.1 Cell culture and generation of knockout cell lines

Mouse ESC culture and the generation of *Asxl2* KO ESCs through CRISPR/Cas9 technique were performed as previously described.^29^ Briefly, proper amount of ESCs were seeded on 0.1% gelatin‐coated plates in GMEM medium (GIBCO) supplemented with 15% fetal bovine serum, 2 mM l‐glutamine, 1× penicillin/streptomycin, 1× non‐essential amino acids, 0.5 mM β‐mercaptoethanol and 100 U/mL leukaemia inhibitory factor. To delete *Asxl2*, the specific sgRNA was designed to target Exon 5 of mouse *Asxl2* and cloned into pX458 vector (Addgene #48138). Then the parental ESCs were transfected with 10 μg of the sgRNA vector with Lipofectamine 3000. The successfully transfected cells were sorted for GFP expression in 48 h after transfection. Around 500 GFP‐positive cells were seeded on 10 cm dishes. After 10 days culture, single colonies were picked and expanded for another 7 days before harvesting for genome DNA extraction. The positive clones were validated by sanger sequencing. As no animal experiments were performed in this study, ethical considerations were not required.

### 2.2 Cardiomyocyte differentiation and mesoderm precursor cells differentiation

The differentiation was performed as described.^30^ For cardiomyocyte (CM) differentiation, ESCs were resuspended in the differentiation medium and cultured in the form of hanging drop (1000 cells/20 μL) on 15 cm dish covers for 2 days. Then the EBs were formed and flushed over and gently resuspended in PBS. After settling down, EBs were transferred to low adsorption dishes and cultured for another 3 days. Then the floating EBs were harvested and seeded on 0.2% gelatin‐coated plates for another 4 days. Beating EBs could be observed under microscope.

For mesoderm precursor cells (MES) differentiation, EBs harvested from dish covers were dissociated after trypsinization. Around 5 × 10^5^ cells were seeded on 0.2% gelatin‐coated 10 cm plates and cultured with differentiation medium containing 10 ng/mL vEGF, 20 ng/mL a‐Activin A, 5 ng/mL BMP4 for another 3 days. Then cells were harvested for RNA extraction or chromatin preparation.

### 2.3 Reverse transcription and quantitative real‐time polymerase chain reaction

Total RNA was extracted with TRIZOL (Invitrogen). Reverse transcription and quantitative real‐time polymerase chain reaction (RT‐qPCR) were performed as described.^29^ Gene expression was determined relative to *RpO* using the ΔCt method. Primer sequences are listed in Table S1.

### 2.4 Chromatin immunoprecipitation

Chromatin preparation was performed as previously described.^31^ First, 1 × 10^7^ cells were crosslinked with 1% formaldehyde for 10 min at room temperature (RT) and then quenched with 0.125 M glycine for another 5 min. Then cells were washed with ice cold PBS and lysed in SDS buffer (100 mM NaCl, 50 mM Tris–HCl pH 8.1, 5 mM ethylene diamine tetraacetic acid [EDTA] pH 8.0, 0.5% SDS, 1× protease inhibitor cocktail [PIC]). After spinning, nuceli were resuspended in appropriate volume of ice‐cold IP buffer (100 mM NaCl, 50 mM Tris–HCl pH 8.1, 5 mM EDTA pH 8.0, 0.3% SDS, 1.0% Triton X‐100) for sonication using a BioRuptor sonicator (Diagenode). After centrifugation at 16,000*g* for 20 min at 4°C, the fragmented chromatin was divided into different aliquots that were incubated overnight with primary antibodies (Table S2) at 4°C. Next, 30 μL protein A/G magnetic beads were incubated with the reaction for another 3 h at 4°C. Then beads were washed three times with high salt buffer (1% Triton X‐100, 0.1% SDS, 500 mM NaCl, 2 mM EDTA pH 8.0, 20 mM Tris–HCl pH 8.0) followed by reversal of the crosslinking. Chromatin mmunoprecipitation (ChIP) DNA was purified for qPCR analysis (Primer sequences are listed in Table S1) or library preparation. Libraries for both ChIP and input samples were prepared using TD503 Kit (Vazyme) according to the manufacturer's instruction. The successfully prepared libraries were sequenced as 150 bp paired‐end reads on Illumina NextSeq 500 platform.

### 2.5 H2AK119ub1 ChIP

For H2AK119ub1 ChIP, pre‐extraction steps were performed before fixation.^31^ In brief, cell pellets were resuspended in cold CSK buffer (100 mM NaCl, 300 mM sucrose, 3 mM MgCl_2_, 10 mM 1,4‐Piperazinediethanesulfonic acid pH 6.8) containing Triton X‐100 (0.5%) and ethylene glycol tetraacetic acid (1 mM) on ice for 5 min. The pre‐extracted cells were then proceeded with regular chromatin preparation. The IP buffer was dialyzed to lower SDS concentration to 0.01% followed by standard IP assays with H2AK119ub1 antibody (CST 8240S). After reversal of the crosslinking, ChIP DNA was purified for qPCR analysis.

### 2.6 H2AK119ub1 CUT&Tag

WT or KO cells were washed twice in wash buffer (20 mM HEPES pH 7.5, 150 mM NaCl, 0.5 mM spermidine, 1× PIC). Meanwhile Concanavalin A beads (Bangs Laboratories, BP531) were activated by washing twice in binding buffer (20 mM HEPES pH 7.5, 10 mM KCl, 1 mM MnCl_2_, 1 mM CaCl_2_), followed by mixing with the cells at RT for 15 min. The supernatant was then removed and beads were resuspended in 100 μL antibody buffer (20 mM HEPES pH 7.5, 150 mM NaCl, 0.5 mM spermidine, 1× PIC; 0.05% digitonin 2 mM EDTA, and 30% BSA). Then 1 μg H2AK119ub1 antibody was added and incubated overnight at 4°C. On the next morning, the supernant containing primary antibody was removed and the beads were incubated in 100 μL of dig‐wash buffer (20 mM HEPES pH 7.5, 150 mM NaCl, 0.5 mM spermidine, 0.0125% digitonin, 1× PIC) containing the secondary antibody at RT for 1 h. Then the pellets were washed in 800 μL dig‐wash buffer for three times. Meanwhile 1:200 dilution of pA‐Tn5 adapter complex (~0.04 μM) was prepared in Dig‐300 Buffer (0.05% digitonin, 20 mM HEPES pH 7.5, 300 mM NaCl, 0.5 mM spermidine, 1× PIC). Then the beads were incubated with pA‐Tn5 at RT for another 1 h. After washing three times by Dig‐300 buffer, the pellets were resuspended in 50 μL tagmentation buffer (10 mM MgCl_2_ in Dig‐300 Buffer) and incubated at 37°C for 1 h. Then stop buffer (2.25 μL of 0.5 M EDTA, 2.75 μL of 10% SDS and 0.5 μL of 20 mg/mL proteinase K) was added and incubated at 50°C for 1 h. To extract DNA from the reaction, 300 μL hydroxybenzene–chloroform–isoamyl alcohol were added, thoroughly mixed and centrifuged for 5 min at 16,000*g*. The aqueous phase was collected and mixed with 300 μL chloroform. After centrifuging for another 5 min at 16,000*g*, aqueous phase was then mixed with 750 μL ethanol overnight at 4°C. The samples were centrifuged for 15 min at 16,000*g* at 4°C. After being washed twice in 1 mL 80% ethanol, the DNA was resuspended in 22 μL TE buffer and used for library amplification (Vazyme).

### 2.7 Immunofluorescence

Cells were seeded onto slides and cultured in ESC medium or differentiation medium. At designated time points, cells were fixed for 10 min with 4% paraformaldehyde. Then the cells were washed in PBS and treated with PBS + 0.5% TritonX‐100 for 5 min. After blocking with 0.5% BSA in PBS for 1 h, cells were incubated at 4°C overnight with antibodies as listed in Table S2. After two washes with PBS‐1% Tween‐20, samples were incubated with secondary antibodies (1:200) in PBS for 2 h, followed by 5 min incubation in DAPI nuclear stain. Images were captured using a DP72 fluorescence microscope.

### 2.8 Chromatin immunoprecipitation sequencing data processing

We uniformly processed the chromatin immunoprecipitation sequencing (ChIP‐seq) and CUT&Tag data according to standard pipelines.^32^, ^33^ In brief, clean reads were aligned to the mouse reference genome assembly GRCm38 (mm10) and only unique mapped reads were used for subsequent analysis. Peaks for histone modifications were called using MACS2^34^ and broad peak calling for H2AK119ub1, while peaks for PcG proteins were called using SPP.^35^ And called peaks were filtered to exclude blacklist regions from ENCODE. Genomic annotation for called peaks was performed using Homer^36^ with default parameters. We considered peak summit within 2.5 kb around TSS of a gene as promoter region and defined peak summit 3 kb away from TSS as non‐promoter region. Normalized coverage track (bigWig) files were generated using bamCoverage from deepTools^37^ with parameters —bs 100—normalizeUsing RPGC—effectiveGenomeSize 2494787188—extendReads 150—scaleFactor and scale factors was determined by ChIPseqSpikeInFree^38^ approach. These normalized profiles were further used to plot heatmaps and metaplots using functions computeMatrix followed by plotHeatmap and plotProfile from deepTools.

### 2.9 Comparison of ChIP‐seq/CUT&Tag signals

To calculate peak intensity, the ChIP‐seq or CUT&Tag signals were extracted from RPGC‐normalized coverage track (bigWig) and averaged according to the top five signal values for each of peaks. To calculate read density in a given region, deepTools was used. Moreover, comparisons between paired ChIP‐seq peak signals at different conditions were performed by MAnorm,^39^ and the *M* value, *p*‐value and read density for each sample were calculated. *M*‐Value indicates log‐transformed fold change of normalized read density between two samples and *p*‐value describes the statistical significance of read intensity difference between the two samples being compared. *M*‐Value was also used to generate boxplot visualization.

To visualize and compare the signals across the genome, we segmented the genome into 2‐kb bins and used the binnedAverage function of GenomicRanges, an R package, to calculate the average density in each bin from the normalized coverage track (bigWig) files. For better visualization, we applied log_2_‐transform to all the signals. Then we used pheatmap, an R package, to cluster the signals and plot the heatmap.

### 2.10 RNA sequencing data processing and analysis

RNA sequencing (RNA‐seq) datasets were performed using the TOPMed RNA‐seq pipeline.^40^ Differentially expressed genes were identified using DESeq2 according to default parameters.^41^ To generate more accurate fold change estimates, lfcShrink function of DESeq2 was used to correct the fold change of lowly expressed genes. Genes with an absolute log_2_ fold change > 1.5 unless otherwise defined and adjusted *p*‐value < 0.05 were considered as differentially expressed ones. ComplexHeatmap^42^ was used to visualize differentially expressed genes. And we performed gene ontology biological process enrichments using clusterProfiler.^43^

### 2.11 Statistical analysis

Statistical analyses were performed using the Student's *t*‐test or Wilcoxon rank sum test, *p*‐values <0.05 was taken as statistically significant.

## 3 RESULTS

### 3.1 Pervasive non‐promoter H2AK119ub1 is associated with RING1B sampling

**FIGURE 1:**
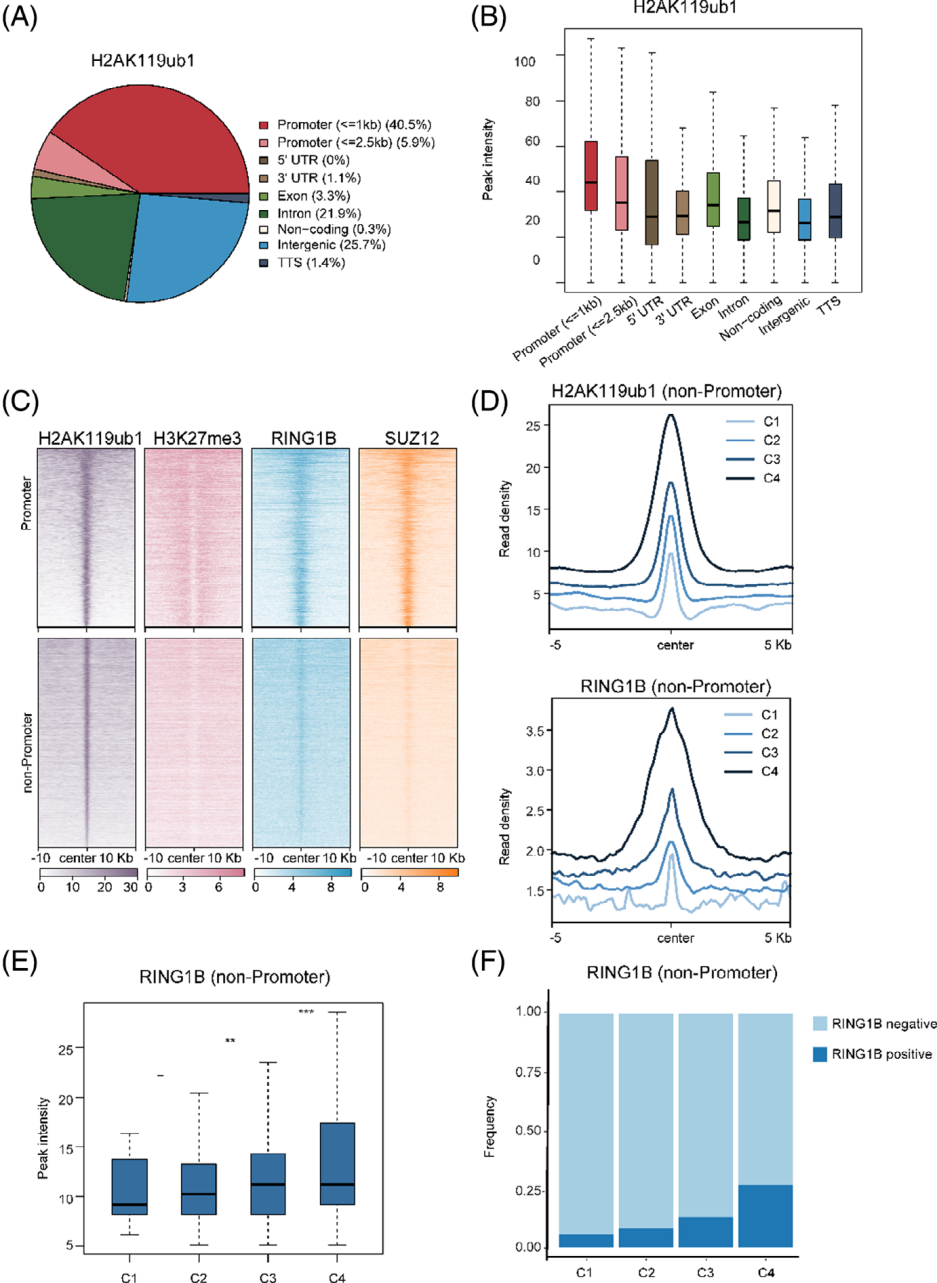
Non‐promoter pervasive H2AK119ub1 is associated with RING1B sampling. (A) Pie plot showing genomic distribution of H2AK119ub1‐occupied regions in mESCs. (B) Boxplots comparing H2AK119ub1 CUT&Tag signals in mESCs across different genomic regions. (C) Heatmaps showing signals for H2AK119ub1 (CUT&Tag), H3K27me3, RING1B and SUZ12 occupancy (ChIP‐seq) at promoters and non‐promoters in mESCs. All rows are centred on H2AK119ub1 peaks and further divided into promoter and non‐promoter clusters. (D) Up: Metaplots showing different H2AK119ub1 levels at non‐promoter regions in mESCs (C1–C4 clusters indicate CUT&Tag signal from low to high). Down: Metaplots showing RING1B ChIP‐seq signal across different levels of H2AK119ub1 clusters. (E) Boxplots comparing RING1B ChIP‐seq signal across C1–C4 clusters (**p value <0.01; ***p value <0.001). (F) Bar plots illustrating the binding frequency of RING1B across different levels of H2AK119ub1 clusters (the binding frequency indicates the proportion of H2AK119ub1 peaks overlapped with RING1B peaks in specific clusters).

Mounting evidences by ChIP‐seq have shown that H2AK119ub1 is pretty abundant and widely distributed throughout the genome. In these assays, cells need to be pre‐extracted to expose the epitope before fixation for ChIP.^7^, ^9^, ^11^, ^31^ To avoid the potential influence of pre‐extraction and fixation, here we performed CUT&Tag analysis for H2AK119ub1. Through comparing H2AK119ub1 enrichment in genome‐wide 2‐kb bins and calculating Pearson correlation coefficiencies, we found that our CUT&Tag data is highly comparable with our own and published ChIP‐seq data in mESCs,^44^ and CUT&Tag data in mouse inner cell mass (ICM)^45^ (Figure S1A). And we noticed that less than half of H2AK119ub1 peaks is enriched at promoters (±2.5 kb around transcription start site [TSS]). Though H2AK119ub1 density is high at promoters, the bulk of H2AK119ub1 is pervasively distributed at intragenic or intergenic regions (Figure 1A,B). And all these signals disappear upon deletion of both *Ring1a* and *Ring1b* in mESCs,^7^ as shown by the heatmap and tracks, indicative of high specificity of the assay. The specificity was further confirmed by an independent ChIP‐qPCR analysis (Figure S1B–D).

In contrast to significant enrichment of H2AK119ub1 at non‐promoter regions as well as promoters, the occupancy of PRC core members RING1B and SUZ12 at non‐promoters is barely detectable (Figure 1C). Nonetheless, when we ranked the H2AK119ub1 levels at non‐promoter regions (four clusters from low to high: C1–C4) and compared the corresponding RING1B densities, we found that they are positively correlated (Figure 1D,E). As relatively low‐density of RING1B peaks could be detected at these regions, we turned to compare the frequency of RING1B binding across the sites with different H2AK119ub1 levels (C1–C4). Interestingly, the frequency of RING1B positive binding is also significantly correlated with H2AK119ub1 densities (Figure 1F). However, poor correlation between residual levels of SUZ12 (C1–C3) and H2AK119ub1 densities is observed (Figure S1E). These data suggests that PRC1 must deposit H2AK119ub1 throughout the genome but is transiently bound at non‐promoter regions where it is not effectively captured by formaldehyde crosslinking, an unavoidable defect in our regular ChIP assay.^46^ Consistently, recent studies by live‐cell tracking showed that majority of PcG proteins diffuse through the nucleus while only a small fraction stably interact with chromatin.^47^, ^48^ Our data indicate that PRC1 sampling frequency or residence time may determine H2AK119ub1 levels, which is worth of further investigation by kinetic studies via single‐molecule imaging techniques. However, it remains unknown what may alter PRC1 sampling or stability at chromatin and thereby fine‐tune H2AK119ub1 levels at weak occupancy sites.

### 3.2 ASXL2 loss results in pervasive non‐promoter accumulation of H2AK119ub1

**FIGURE 2:**
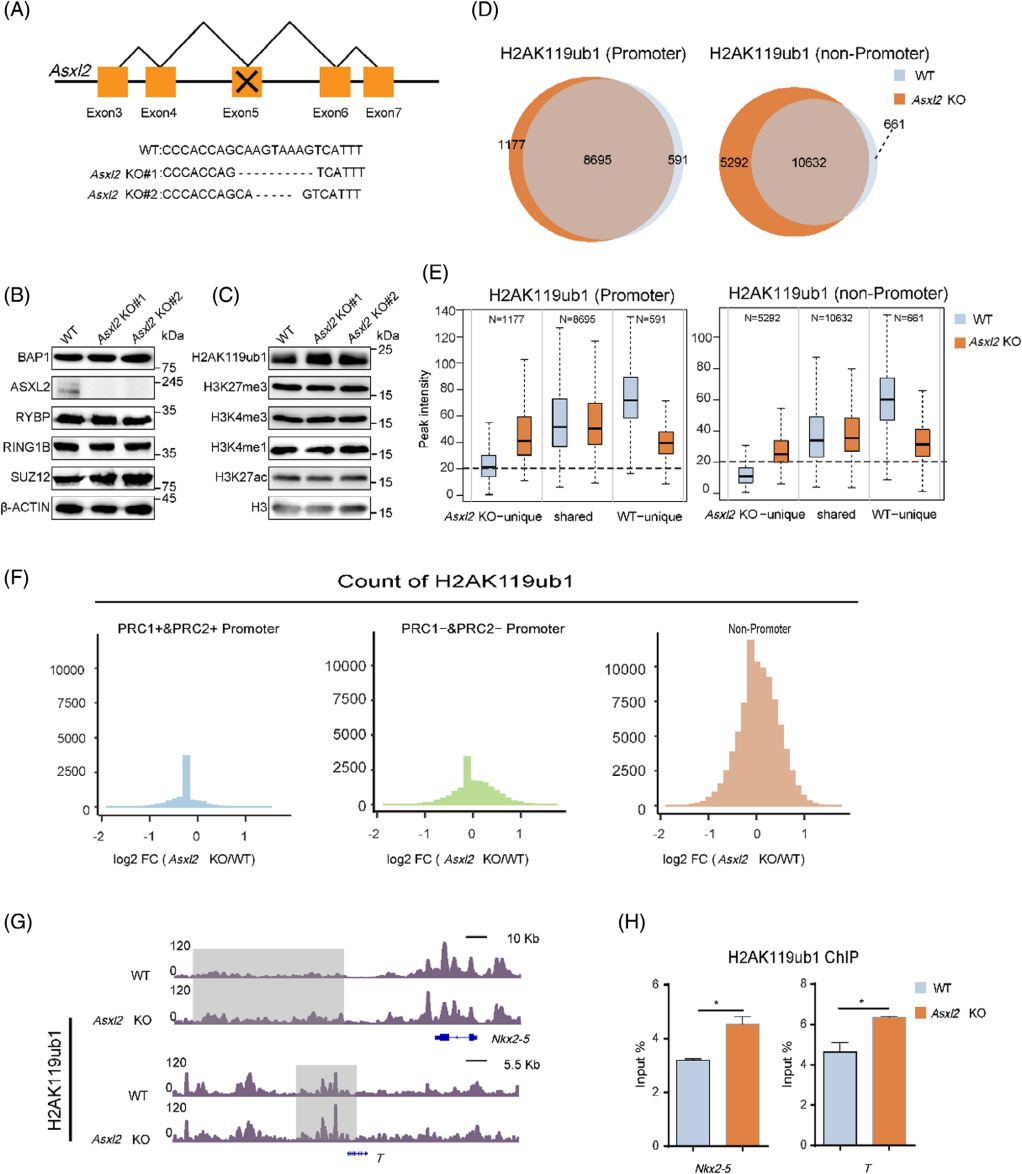
ASXL2 loss results in pervasive gain of H2AK119ub1 at non‐promoter regions. (A) Genome typing of *Asxl2* deletion (two clones) by CRISPR/Cas9 techniques. (B,C) WB assays to compare the levels of designated proteins or histone modifiations in WT and Asxl2 KO mESCs. (D) Venn diagrams showing the overlap of H2AK119ub1 peaks (*q*‐value <0.1) between WT and *Asxl2* KO mESCs at promoter and non‐promoter regions. (E) Boxplots comparing H2AK119ub1 signals of WT and *Asxl2* KO among WT‐unique, shared and *Asxl2* KO‐unique groups at promoter and non‐promoter regions. (F) Histogram plots comparing the log_2_ fold change of H2AK119ub1 signals in Polycomb/non‐Polycomb promoter and non‐promoter groups between *Asxl2* KO and WT mESCs. The H2AK119ub1 signals are the average read densities of each 2‐kb bin. (G) Genome browser view of H2AK119ub1 profile in WT and *Asxl2* KO mESCs. (H) H2AK119ub1 ChIP‐qPCR analysis of designated non‐promoter regions near to two PcG target genes *T*, *Nkx2‐5* (as illustrated in G) in WT and *Asxl2* KO mESCs. Data are represented as the mean ± *SD* of replicates (*n* = 3) (*p < 0.05 and two‐tailed unpaired *t* test).

It has been demonstrated that PR‐DUB deficiency results in overall increase of H2AK119ub1 levels in independent models.^12^, ^13^, ^16^, ^26^, ^27^ Here we would like to find out how H2AK119ub1 would be accumulated at the genome wide upon PR‐DUB inactivation. As deregulated expression of a huge amount of genes was observed in *Bap1*‐knockout (KO) mESCs,^16^ the H2AK119ub1 changes may be secondary to transcription alteration. Thus, we generated *Asxl2*‐KO mESCs. The positive mESC clones of *Asxl2* deletion were verified by targeted genome sequencing and Western blot (WB) assays (Figure 2A,B). Loss of ASXL2 in mESCs does not affect cell morphology, alkaline phosphatase (ALP) activity, or the expression of pluripotency marker genes OCT4 and NANOG (Figure S2A,B). Thus, ASXL2 is not required for mESC self‐renewal.

Consistent with the recent measurement by mass spectrometry (MS),^16^ we found that ASXL2 loss results in modest increase of bulk H2AK119ub1 levels (Figure 2C), though it does not affect the expression levels of core members of PRC1 or PRC2, or other tested histone modification levels (Figure 2B,C). CUT&Tag analysis demonstrates that a larger fraction of H2AK119ub1 is distributed at non‐promoter regions in *Asxl2*‐null mESCs than in WT mESCs (Figure S3A). According to peaks identified by MACS2 (*q*‐value < 0.1), 1177 and 5292 unique H2AK119ub1 peaks emerge respectively from promoters and non‐promoters (Figure 2D). When comparing the peak intensity, we found that the gain of H2AK119ub1 in *Asxl2*‐null mESCs mainly occurs at non‐promoter regions (mainly introns and intergenic regions), where there exists low or no signals in WT mESCs (Figure 2E, Figure S3B,C). To prevent the possible inaccuracy of calling weak peaks, we directly compared average read density of H2AK119ub1 signals at Polycomb/Non‐Polycomb promoters and non‐promoters between *Asxl2* KO and WT mESCs. As shown by the Histogram plots, the increase is mainly observed at non‐promoter regions and a small subset Non‐Polycomb promoters (Figure 2F). Consistently when comparing the peaks distribution, we found that the unique H2AK119ub1 peaks in *Asxl2*‐null mESCs are mainly deposited on PRC1/2‐negative regions (Figure S3D). Furthermore, the increase was validated by independent H2AK119ub1 ChIP‐qPCR analysis at intergenic regions closed to typical PcG target genes (Figure 2G,H). Though no significant chromatin binding of ASXL2 or BAP1 has been successfully detected by ChIP‐seq assay in our hands, these data suggest that PR‐DUB may widely prevent H2AK119ub1 accumulation, especially at PRC‐negative or weak occupancy sites.

### 3.3 Pervasive non‐promoter H2AK119ub1 accumulation in *Asxl2*‐null mESCs is associated with RING1B redistribution

**FIGURE 3:**
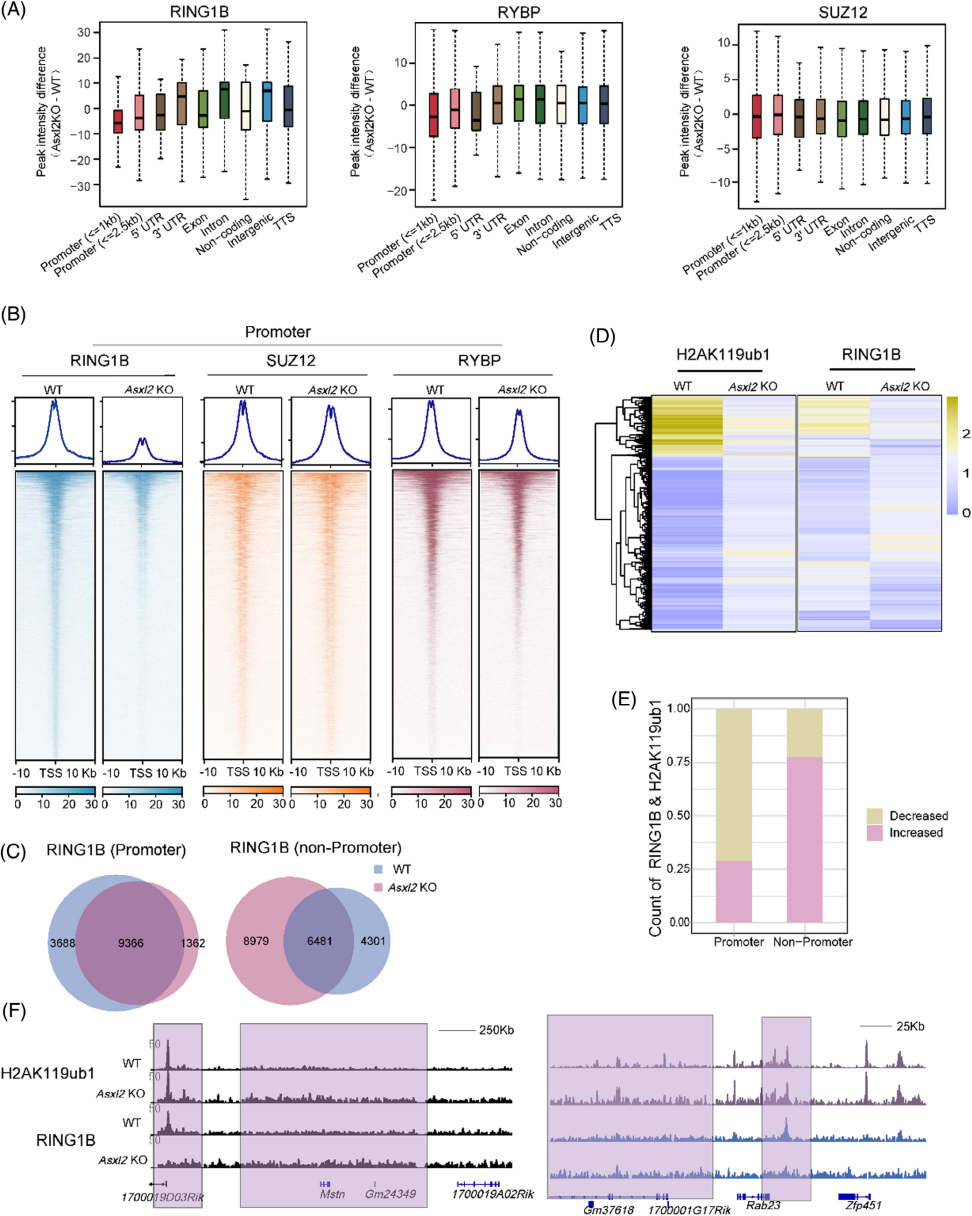
RING1B redistribution is associated with H2AK119ub1 gain at non‐promoter regions in *Asxl2*‐null mESCs. (A) Boxplots comparing differences of RING1B, RYBP and SUZ12 ChIP‐seq signals at defined genomic regions in WT and *Asxl2* KO mESCs. (B) Heatmaps illustrating RING1B, SUZ12 and RYBP ChIP‐seq signals at promoter regions in WT and mESCs. (C) Venn diagrams showing the overlap of RING1B peaks (*q*‐value<0.1) between WT and *Asxl2* KO mESCs at promoter (left) and non‐promoter (right) regions. (D) Heatmap showing the RING1B signal changes simultaneously as H2AK119ub1 signals decrease or increase in *Asxl2* KO versus WT mESCs. The signals are the average read densities of each 2‐kb bin. (E) Stacked bar plot showing the proportion of simultaneous decrease and increase groups as outlined in (D) at promoter and non‐promoter regions. (F) Snapshots of both H2AK119ub1‐ and RING1B‐gained loci at promoter and non‐promoter regions.

To further find out whether and how ASXL2 loss affects PRC deposition, we preformed ChIP‐seq analyses for RING1B, RYBP and SUZ12 in WT and *Asxl2* KO mESCs. As shown in Figure 3A,B, ASXL2 loss causes mild decrease of RYBP and SUZ12 enrichment levels at promoters, while it seems not to significantly affect their densities at non‐promoter regions. In contrast, RING1B densities are significantly increased at non‐promoters accompanied with striking loss from promoters (Figure 3A,B and S4A). Peak calling (*q*‐value<0.1) showed that a large number of RING1B peaks are gained from non‐promoters while lost from promoters (Figure 3C), though the gained peaks are generally low (Figure S4B). Actually, RING1B binding levels are decreased at highly occupied sites (even for the shared peaks, mainly at promoters) and modestly increased at weakly occupied sites in *Asxl2* KO ESCs (Figure S4B). This seemingly redistribution is consistent with the globally unchanged total and chromatin‐bound fraction of RING1B (Figures 2B and S4C). Hence ASXL2 is indispensable for robust PRC1 deposition.

Then we followed to sort out whether there is a correlation between H2AK119ub1 diffusion and RING1B redistribution. Interestingly, H2AK119ub1 levels are significantly increased at sites with newly acquired RING1B peaks in *Asxl2* KO mESCs (*Asxl2* KO unique, mainly at non‐promoters), while remain unchanged at regions without RING1B redistribution (Figure S4D). To avoid the bias of calling peaks at weakly occupied sites, we also compared the signals of PcG proteins and H2AK119ub1 by their average read densities in 2‐kb bins. As shown by the heatmap, most of RING1B signals change simultaneously as H2AK119ub1 signals decrease or increase between *Asxl2* KO and WT mESCs. And the decrease of their signals is mainly observed at promoters, while increase mainly at non‐promoters (Figure 3D,E), though it remains unclear whether the modest gain of RING1B is due to the potential antagonism of H2AK119ub1 against cPRC1.^28^ In contrast, there lacks coincidence of SUZ12 and H2AK119ub1 signal changes between *Asxl2* KO and WT mESCs (Figure S4E). Examples of these coordinated increase of RING1B and H2AK119ub1 densities at non‐promoters and accompanied RING1B loss from promoters are shown in Figure 3F. Together, these data suggest that PR‐DUB deficiency results in excessive accumulation of H2AK119ub1 at non‐promoter regions and corresponding PRC1 redistribution.

### 3.4 RING1B loss from promoters impairs gene silencing in *Asxl2*‐null ESCs

**FIGURE 4:**
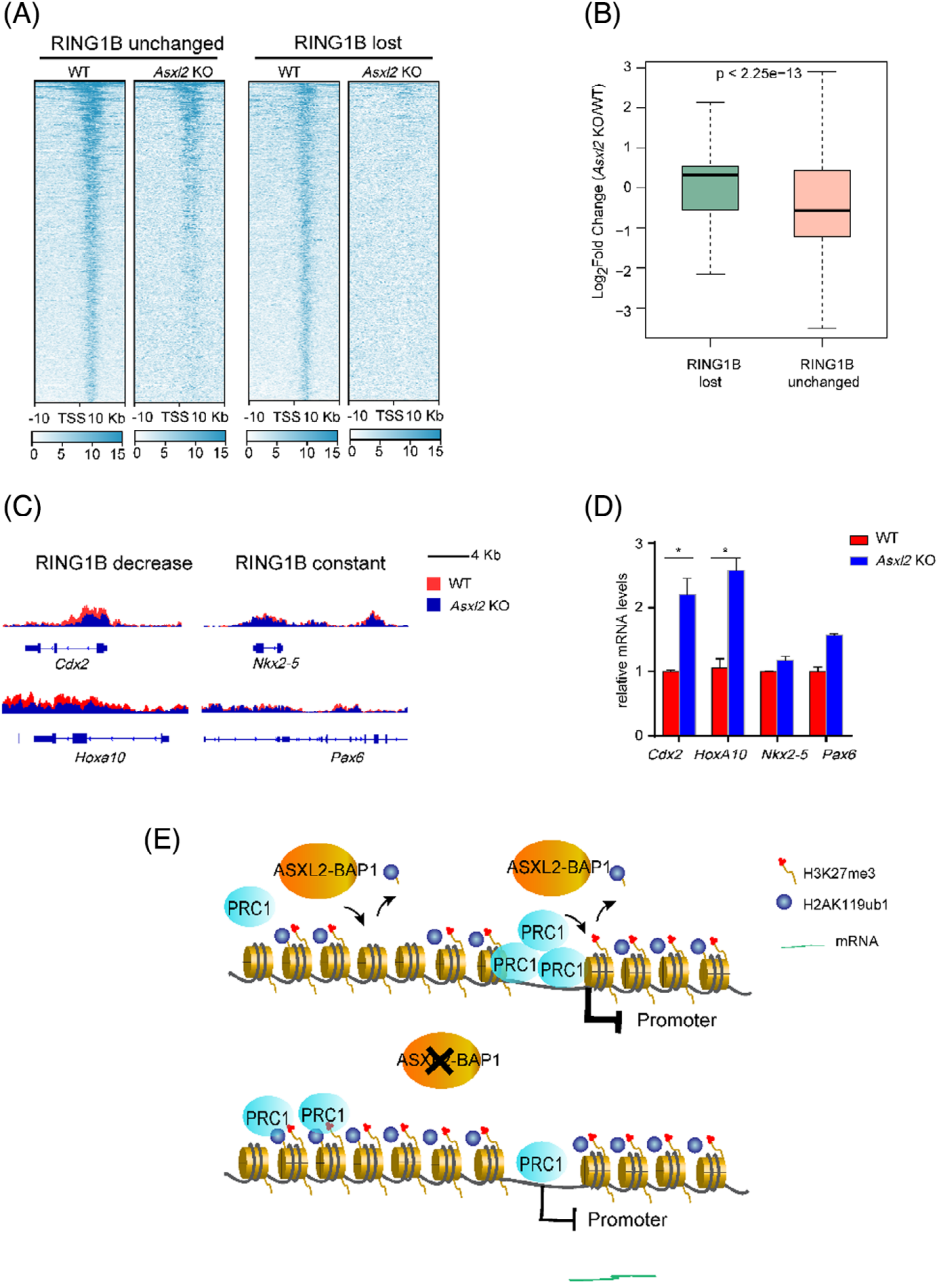
RING1B loss from promoters is associated with impaired gene silencing in *Asxl2*‐null mESCs. (A) Heatmaps illustrating RING1B ChIP‐seq signal at the promoters of with the RING1B binding unchanged (3049 peaks, log_2_ fold change between −0.5 and 0.5) or lost (3316 peaks, log_2_ fold change < −1.5) in *Asxl2* KO mESCs versus WT mESCs. All rows are centred on TSS. (B) Boxplots comparing log_2_ fold change of gene expression in *Asxl2* KO mESCs versus WT mESCs between RING1B‐unchanged and RING1B‐lost clusters. (C) Snapshots comparing RING1B signals at the promoters of designated genes in WT and *Asxl2* KO mESCs. (D) RT‐qPCR analysis of mRNA levels of designated genes in WT and *Asxl2* KO mESC. Data are represented as the mean ± *SD* of replicates (*n* = 3) (*p < 0.05 and two‐tailed unpaired *t* test). (E) Model for PR‐DUB in safeguarding PcG functions. Normally robust deposition of PRC1 is restricted to target promoters though its sampling may produce low H2AK119ub1 levels at the genome wide. Upon PR‐DUB deficiency, the pervasive gain of H2AK119ub1 at non‐promoters is associated with titration of PRC1 away from target promoters and thereby leads to compromised maintenance of gene silencing.

Considering of the significant loss of RING1B from promoters, we queried the gene expression profiles through RNA‐seq analysis between WT and *Asxl2*‐null mESCs. Consistent with the unaffected mESC self‐renewal by ASXL2 loss (Figure S2), pretty minor deregulation of gene expression is observed (45 genes upregulated and 172 genes downregulated with log_2_ fold change >1.5 or less than −1.5, respectively). Nevertheless, compared with the promoters with unchanged RING1B binding (3316 peaks, log_2_ fold change [−0.5, 0.5]), the ones with strong decrease of RING1B enrichment (3049 peaks, log_2_ fold change < −1.5) in *Asxl2* KO cells are associated with mild but significant gene derepression (Figure 4A,B). For examples, RING1B peaks were decreased at the promoters of *Cdx2* and *Hoxa10* while remain unchanged at the promoters of *Nkx2.5* and *Pax6* in *Asxl2* KO ESCs (Figure 4C). Accordingly, we examined their gene expression levels by RT‐qPCR analysis. As shown in Figure 4D, the mRNA levels of Cdx2 and Hoxa10 are specifically upregulated in *Asxl2* KO cells. Hence undermined RING1B deposition at promoters at the absence of ASXL2 affects the robust maintenance of target gene silencing.

Taken together, these data indicate that PR‐DUB monitors and restricts H2AK119ub1 at the genome wide. Upon delimitation from PR‐DUB, increased H2AK119ub1 activity at non‐promoters is associated with favourable RING1B occupancy, which is consistent with the concept of chromatin sampling.^49^ Accordingly, PRC1 is diluted from repressed promoters, leading to impaired gene silencing. In this scenario, PR‐DUB indeed acts as a PRC despite of an opposite biochemical activity against PRC1 (Figure 4E).

### 3.5 ASXL2 loss impairs PcG repression and lineage differentiation

Considering that PRCs play critical roles in lineage specification, we sought to examine how ASXL2 loss would affect ESC differentiation. Given that ASXL2 is expressed at high levels in heart and *Asxl2*‐null mice exhibit impaired heart function,^50^ we first set up a CM differentiation model (Figure S5A). After embryoid body (EB) formation for 5 days, *Asxl2*‐deleted EBs are significantly larger and more compacted than the WT counterparts, indicative of an early defect of germ layer differentiation. At day 9 of differentiation, almost all control EBs are beating, while only around 30% of *Asxl2* KO EBs contain beating clusters (Figure S5B,C). Furthermore, immunofluorescence analysis of the cardiac‐mark gene alpha‐ACTIN shows a significantly lower expression in the derived cells from *Asxl2*‐KO EBs (Figure S5D,E) than the WT EBs. Harvesting samples from several time points of differentiation, we compared the expression of distinct lineage genes. As shown in Figure S5E, the downregulation of pluripotency gene expression is significantly blocked by Asxl2 deletion. For the different germ layer genes in *Asxl2* KO cells, the expression of mesoderm and endoderm lineage genes is less efficiently induced while the expression of ectoderm lineage genes is overactivated, compared with the controls. To compare this phenotype with PcG mutants, we similarly took advantage of a previously generated *Ring1b* catalytic inactive mutant ESC line (*Ring1b* ^I53A^)^7^ for CM differentiation. Interestingly, RING1B inactivation also significantly affects EB formation and the generation of beating cardiac lineage cells (Figure S5B–D). These data suggest that ASXL2 as well as PRC1 activity is required for the formation of germ layer and cardiac lineage differentiation.

**FIGURE 5:**
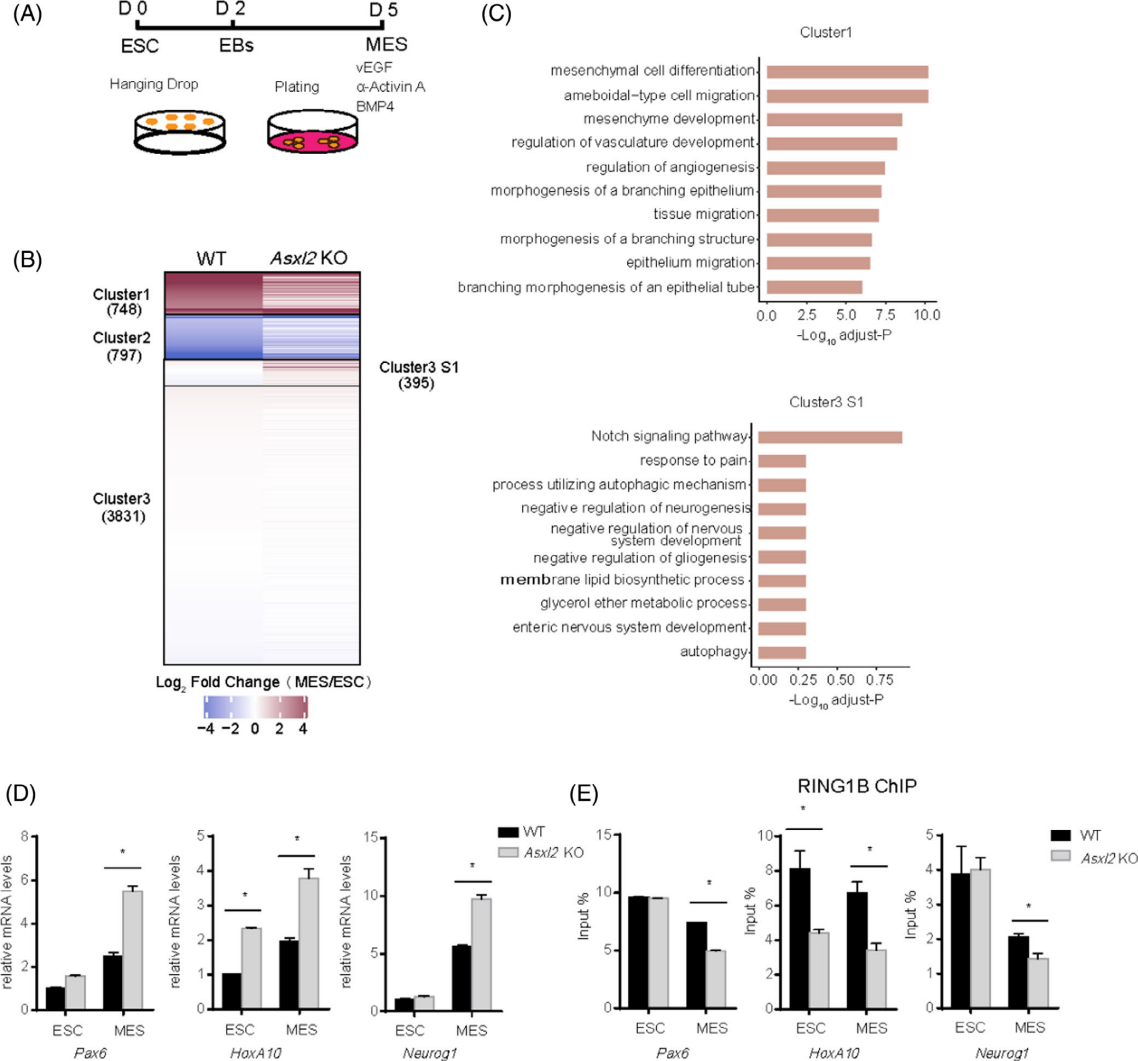
ASXL2 loss results in aberrant lineage differentiation. (A) Schematic diagram showing differentiation strategy of ESC to MES. (B) Heatmap illustrating the proportion of genes with up‐ and down‐regulation according to log_2_ fold change of gene expression between WT‐MES and WT‐ESC, Cluster 1 (WT log_2_ fold change > 3), Cluster 2 (WT log_2_ fold change < −3), Cluster 3 (WT −0.5 < log_2_ fold change<0.5). (C) GO enrichment analysis of genes in Cluster 1 and Cluster 3 S1, top 10 enriched items are shown according to –log_10_ adjusted‐*p*. (D) RT‐qPCR analysis of the mRNA levels of non‐MES lineage genes *Pax6*, *Hox10* and *Neurog1* in designated groups of cells. (E) ChIP‐qPCR analysis of RING1B binding at the promoters of *Pax6*, *Hox10* and *Neurog1* in designated groups of cells. Data are represented as the mean ± *SD* of replicates (*n* = 3) (*p < 0.05 and two‐tailed unpaired *t* test for D and E).

To get a high‐quality dataset of transcriptome profiling, we followed an optimized protocol that derives a highly homogenous MES^51^ (Figure 5A). To monitor the dynamic changes of gene expression during MES differentiation upon *Asxl2* loss‐of‐function, we performed RNA‐seq analysis at day 0 and day 5 of differentiation with both WT and *Asxl2* KO groups. Based on the differentially expressed genes during differentiation of WT cells, we subcategorized them to three clusters: Cluster 1 defines upregulated genes in MES, Cluster 2 downregulated genes in MES and Cluster 3 unchanged (Figure 5B). For Cluster 1 genes, ASXL2 loss significantly affect the activation of 748 genes (Figure 5C). Gene ontology (GO) analysis demonstrates that these genes are mainly enriched in mesenchyme development and heart morphogenesis, etc (Figure 5C). RT‐qPCR analysis confirmed that the activation of *T* (Brachyury) and *Nkx2.5*, respectively a mesoderm and cardiac lineage development regulator gene, is negatively affected by ASXL2 loss (Figure S5F). Thus, ASXL2 is required for the activation of lineage specific genes during differentiation.

Focusing on the genes of Cluster 3 which remains inactive during differentiation in WT group, we noticed a small subcluster of genes that are prematurely activated (Cluster 3 subcluster 1, C3S1, 395 genes) in *Asxl2* KO MES (Figure 5B). GO analysis shows that this subgroup of genes is linked to Notch signal‐pathway and nervous system development (Figure 5C). RT‐qPCR analysis confirms that ASXL2 loss results in significantly higher expression of neuroectoderm genes such as *Pax6*, *Hoxa10* and *Neurog1* (Figure 5D). Therefore, ASXL2 loss leads to untimely activation of non‐lineage specific genes. Considering that these genes are typical PcG targets, we performed RING1B ChIP‐qPCR analysis at their promoters. We found that RING1B binding is significantly decreased in *Asxl2* KO MES, though yet unchanged in *Asxl2* KO ESCs compared with the WT counterparts (Figure 5E). It suggests that PR‐DUB exerts spatiotemporal effects on safeguarding PcG deposition at promoters during differentiation. Collectively, these data indicate that PR‐DUB deficiency impairs PcG repression and leads to improper lineage specification.

## 4 DISCUSSION

In the past decades, great efforts have been put into identification of writers and erasers for chromatin modifications and characterization of their functions. Biochemically these modifiers antagonize each other, however they exert more complexed regulatory roles than expected. Here we demonstrate that PR‐DUB and PRC1 coordinate for chromatin occupancy and H2AK119ub1 activity in transcription regulation.

It is now well recognized that PcG engages with chromatin and stabilizes the repressive chromatin environment.^2^, ^4^, ^52^ Meanwhile emerging studies have shown that PcG proteins are far more dynamic than unexpected.^1^, ^4^, ^47^, ^48^, ^53^ We and others have demonstrated that PRC2 and ncPRC1.1 select their target sites by sampling inactive CpG islands, usually at promoters.^49^, ^54^, ^55^ This sampling module allows PRCs to engage all potential target promoters in the genome while only to achieve stable binding at favourable repressive environment through formation of PcG domain or condensates.^1^, ^4^, ^53^ In agreement with this framework, we have observed the coincidence between redistributed RING1B and H2AK119ub1 (Figure 3). The bulk of H2AK119ub1 diffused outside of bivalent promoters is likely produced by sampling PRC1 while limited by PR‐DUB. When PR‐DUB is deficient, roaming ncPRC1 (probably PCGF3/5‐PRC1 as suggested^27^) is delimited at weak occupancy sites to gain H2AK119ub1. However, it remains unknown how the counterbalance between PRC1 and PR‐DUB is spatiotemporally achieved.

Another interesting finding in our study is that RING1B is titrated away from canonical bivalent promoters in *Asxl2*‐null mESCs, accompanied with diffusely gained chromatin occupancy of RING1B. Probably because of constant levels of PcG proteins, the increased occupancy of PRCs at cryptic regions is usually coincident with the decreased enrichment at strong target sites.^56^, ^57^ Notably, even PRC2 loss from promoters in *ASXL1*‐mutated leukaemia cells^18^ or in *Bap1*‐null mESCs^26^, ^27^ have been reported, which could be secondary to gene derepression. In our data, the specifically observed RING1B redistribution should be a direct consequence, as ASXL2 loss in mESCs does not significantly affect cell fate or gene expression like *Bap1* deletion.^26^, ^27^ Nevertheless, direct evidence of re‐targeting of catalytically active PRC1 still lacks. The PRC1 sampling does not seem to be attributed to the H2AK119ub1 reader RYBP, as we failed to establish the association between H2AK119ub1 and RYBP at non‐promoter regions (Figure 3). Recently it has been suggested that PCGF3/5‐PRC1 mainly accounts for the addition of non‐promoter H2AK119ub1.^27^, ^58^ Given that PCGF3/5‐PRC1 contains its unique DNA binding factors and potential RNA binding capabilities,^58^, ^59^, ^60^, ^61^ it may keep scanning or sensing DNA or RNA. Given that *ASXL1/2* and *BAP1* are frequently mutated in human malignancies,^62^, ^63^ further investigation is warranted to find out whether and how RING1B redistribution contributes to tumorigenesis.

It is also worth noting that H2AK119ub1 has been recently found to be associated with transcription responsiveness.^64^, ^65^, ^66^ And PR‐DUBs have been demonstrated to function as transcriptional activators through counteracting H2AK119ub1 at promoters,^12^, ^16^, ^25^, ^67^ or maintaining enhancer functions.^17^, ^68^, ^69^ Hence knowledge gaps still remain to be filled to resolve these discrepancies. In this study, we focus on the roles of H2AK119ub1 regulation during ESC differentiation. Actually, H2AK119ub1 distribution is highly dynamic in early mouse embryos.^45^ At pre‐implantation stage, the first cell fate decision leads to the differentiation of trophectoderm (TE) and ICM.^70^ So far, the H2AK119ub1 profiles in TE is not clear. And TE specification from ESC is rather difficult, which requires reprogramming of chromatin states and transcription regulatory network.^71^, ^72^ Thus, it will be interesting to find out whether how PRC1 and PR‐DUB regulates TE specification through dynamic control of H2AK119ub1 in human and mouse.

In sum, our study has demonstrated that PR‐DUB restricts PRC1‐mediated H2AK119ub1 diffusion on genome and prevents cryptic PRC1 deposition to safeguard PcG repression at promoters. In a broad sense, we provide a paradigm that biochemically antagonistic chromatin modifiers may functionally assist each other for transcription regulation. This study also provides mechanistic insights into derailed epigenetic regulatory network that may be targeted in relevant cancers.

## AUTHOR CONTRIBUTIONS

Xudong Wu and Mulin Jun Li conceived and designed the research. Rui Li and Yingying Zhao performed most of the experiments and part of the bioinformatic analysis. Dandan Huang and Ye Yuan performed computational analysis. Dawei Huo, Zhongye Dai, Xiaoyu Sun and Xiaozhi Liu assisted with experiments. Kristian Helin provided the ASXL2 antibody. All authors contributed to the writing of the manuscript, read and approved the final manuscript.

## CONFLICT OF INTEREST STATEMENT

The authors declare no conflict of interest.

## Supporting information


**Appendix S1.** Supporting Information.

## Data Availability

All datasets generated in this work have been deposited to Gene Expression Omnibus (GEO) under accession number GSE186375.
