# Supplementary material for: PR‐DUB safeguards Polycomb repression through H2AK119ub1 restriction
Source: Cell Prolif. 2023 Mar 23;56(10):e13457. doi: 10.1111/cpr.13457 (PMC10542648; doi:10.1111/cpr.13457)
Supplement: Supplementary file 1 — Appendix S1. Supporting Information. [file CPR-56-e13457-s001.docx]

**PR-DUB safeguards Polycomb repression through H2AK119ub1 restriction**

Rui Li, Dandan Huang, Yingying Zhao, Ye Yuan, Xiaoyu Sun, Zhongye Dai, Dawei Huo, Xiaozhi Liu, Mulin Jun Li, Xudong Wu

**Supplementary information**

**Figures (Figures. S1-S5).**

**Table S1. Primer sequences for qPCR and ChIP-qPCR analysis.**

**Table S2. Antibodies Used in this Study.**

**Figures and figure legends**

**
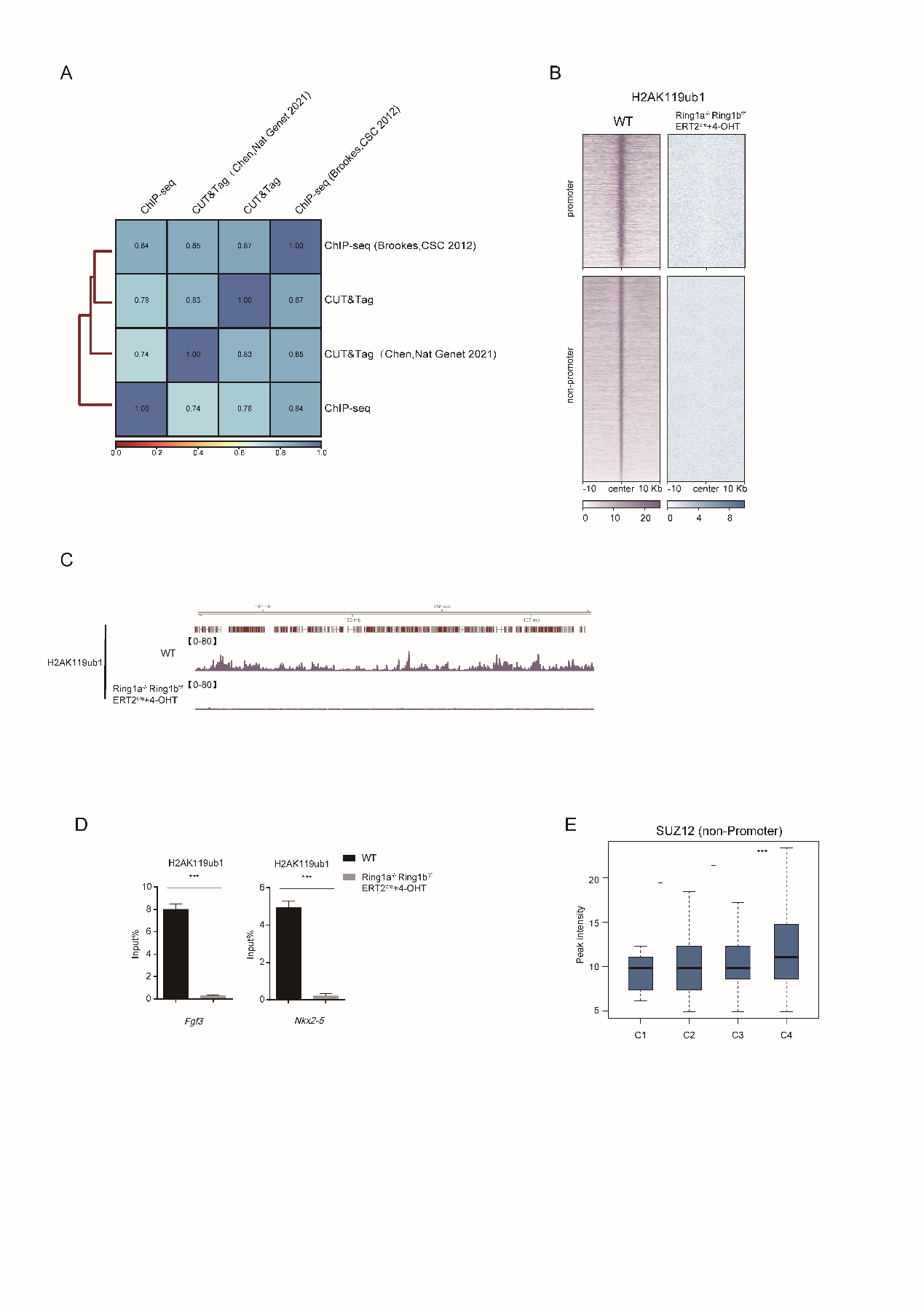
**

**Figure S1 Experimental control. A**. Genome-wide Pearson correlation (2-kb bins) of H2AK119ub1 enrichment (RPKM) in our own or published CUT&Tag or ChIP-seq datasets. **B**. Heatmaps illustrating H2AK119ub1 CUT&Tag signal in WT and *Ring1a*-/-*Ring1b*f/f^ERT2cre^+4-OHT (*Ring1a/b* double knockout) mESCs. All rows are centered on H2AK119ub1 peaks. **C**. Snapshots of H2AK119ub1 peaks in WT and *Ring1a/b* double knockout mESCs. **D**. ChIP-qPCR analysis of H2AK119ub1 levels at the promoters of *Fgf3*, *Nkx2-5* in WT and *Ring1a/b* double knockout mESCs. **D**. Boxplots comparing SUZ12 ChIP-seq signal across different levels of H2AK119ub1 clusters (****P*<0.001).


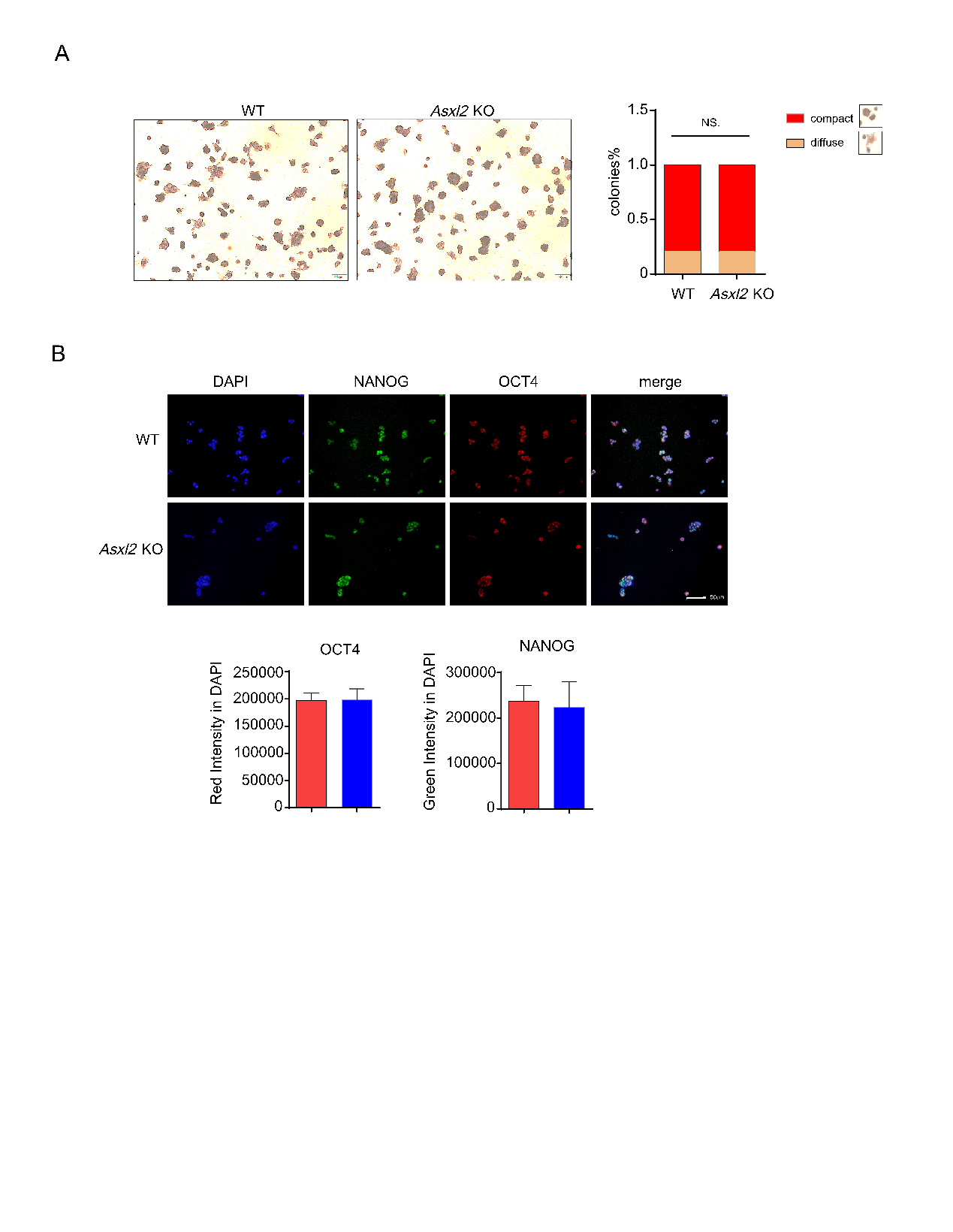


**Figure S2 Characterization of *Asxl2* KO mESCs. A**. Left: ALP staining of WT and *Asxl2* KO mESCs. Right: The statistics of percentage of compact or diffuse colonies in WT and *Asxl2* KO mESCs. **B**. IF staining of NANOG and OCT4 in WT and *Asxl2* KO mESC. The scale bar represents 100 μm. The column shows relative density of OCT4 and NANOG-positive cells. NS., non-significant.

**
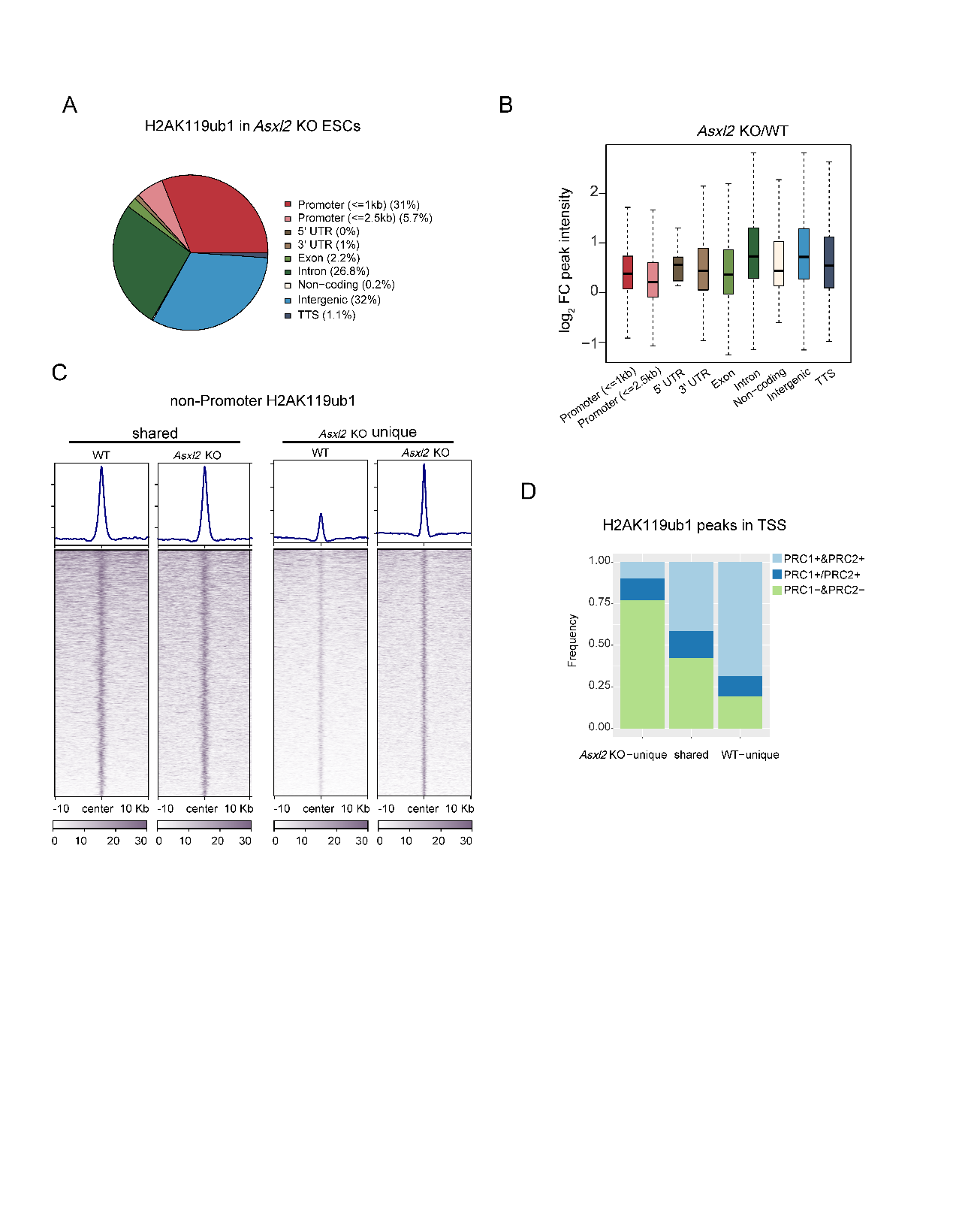
**

**Figure S3 The deregulated H2AK119ub1 in *Asxl2* KO mESCs. A**. Pie plot showing H2AK119ub1 ChIP-seq signals in *Asxl2* KO mESCs across different genomic features. **B**. Boxplots comparing log_2_ fold change of H2AK119ub1 ChIP-seq signals between *Asxl2* KO and WT mESCs. **C**. Heatmaps illustrating non-promoter H2AK119ub1 signal of shared and *Asxl2* KO-unique groups in WT and *Asxl2* KO mESCs. **D**. Bar plots comparing the proportion of H2AK119ub1 peaks at promoters overlapped with PRC1 (RING1B) and/or PRC2 (SUZ12)-occupied genes across different H2AK119ub1-positive groups (WT-unique, shared or *Asxl2* KO-unique).


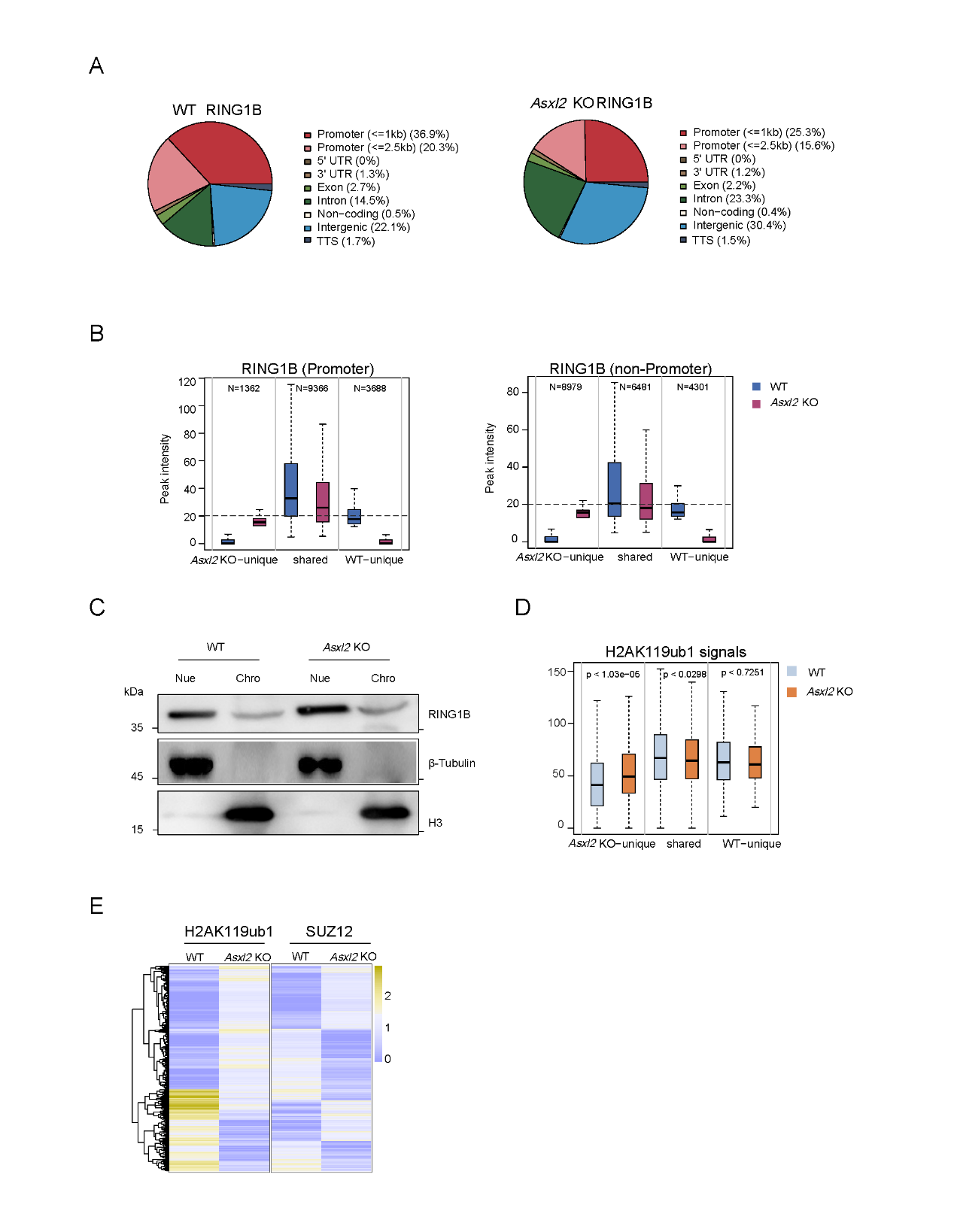


**Figure S4 The deregulated RING1B occupancy in *Asxl2* KO mESCs. A**. Pie plots comparing RING1B ChIP-seq signals in WT (Left) and *Asxl2* KO (Right) mESCs across different genomic features. **B**. Boxplots comparing RING1B ChIP-seq signal among WT-unique, shared and *Asxl2* KO-unique groups in WT and *Asxl2* KO mESCs at promoter (Left) and non-promoter (Right) regions. **C**. Western blot comparing indicated protein levels in nucleoplasmic or chromatin fractions. β-Tubulin and H3 respectively serve as loading controls for each fraction. **D**. Boxplots comparing H2AK119ub1 CUT&Tag signal among different RING1B-binding groups (WT-unique, shared and *Asxl2* KO-unique). **E**. Heatmap showing the changes of SUZ12 and H2AK119ub1 signals in *Asxl2* KO versus WT mESCs. The signals are the average read densities of each 2-kb bin.


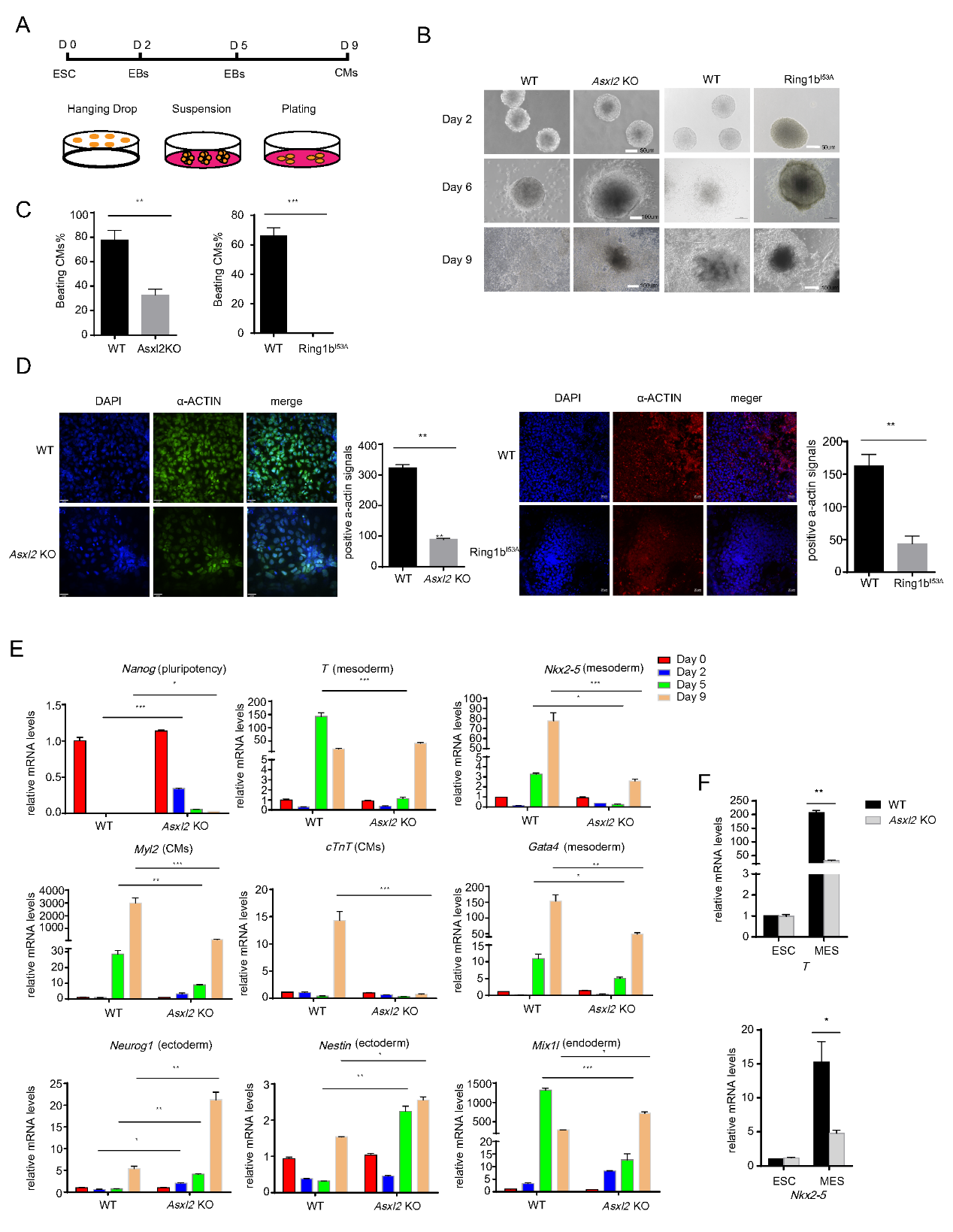


**Figure S5 ASXL2 loss results in aberrant cardiomyocyte differentiation. A**. Schematic diagram shows differentiation strategy of ESC to CMs. **B**. Bright-field shows EBs at Day 2, 5, 9 of differentiation. The scale bars represent 50 μm at Day 2 and 100 μm at Day 5 and 9 of differentiation. **C**. Percentage of beating EBs at day 9 of differentiation. Data are represented as the mean ± SD of replicates (n=3) (* **P*<0.01, ****P*<0.001, and two-tailed unpaired *t* test). **D**. IF staining of α-ACTIN both in WT, *Asxl2* KO or *Ring1b*^I53A^-CMs. The column shows relative α-ACTIN signals. Data are represented as the mean ± SD of replicates (n=3) (** *P*<0.01, and two-tailed unpaired *t* test). **E-F.** RT-qPCR analysis of expression levels of lineage genes in designated groups of cells. Data are represented as the mean ± SD of replicates (n=3) (**P*<0.5, ***P*<0.01, ****P* <0.001, and two-tailed unpaired *t* test).

**Table S1. Primer sequences for qPCR and ChIP-qPCR analysis .**

| **NAME** | **SEQUENCE** | **Assay** |
| --- | --- | --- |
| Hoxa10-F | CCTGCCGCGAACTCCTTTT | qRT-PCR |
| Hoxa10-R | GGCGCTTCATTACGCTTGC | qRT-PCR |
| PAX6-F | ACTTGGACGGGAACTGACAC | qRT-PCR |
| PAX6-R | AACAACCTGCCTATGCAACC | qRT-PCR |
| Cdx2-F | CAGCCGCCGCCACAACCTTCCC | qRT-PCR |
| Cdx2-R | TGGCTCAGCCTGGGATTGCT | qRT-PCR |
| NkX2.5-F | TGCAGAAGGCAGTGGAGCTGGACAAGCC | qRT-PCR |
| NkX2.5-R | TGCACTTGTAGCGACGGTTCTGGAACCAG | qRT-PCR |
| Neurog1-F | CGATCCCCTTTTCTCCTTTC | qRT-PCR |
| Neurog1-R | TGCAGCAACCTAACAAGTGG | qRT-PCR |
| mT-F | TGCACATTACACACCACTGACG | qRT-PCR |
| mT-R | AGAACCAGAAGACGAGGACGTG | qRT-PCR |
| mNANOG-F | GCTTTGAAGCATCCGACTGTA | qRT-PCR |
| mNANOG-R | TCTTGACCGGGACCTTGTCT | qRT-PCR |
| mGata4-F | TTCCTCTCCCAGGAACATCAAA | qRT-PCR |
| mGata4-R | GCTGCACAACTGGGCTCTACTT | qRT-PCR |
| mNestin-F | AGAGGAAGAGCAGCAAGGCCATGAC | qRT-PCR |
| mNestin-R | TCCCTGACTCTGCTCCTTCTTCTTCAT | qRT-PCR |
| Myl2-F | CAACGGCTGCATCAACTATG | qRT-PCR |
| Myl2-R | GGCCAGGAAAGACTACCACA | qRT-PCR |
| CTnT-F | AAAATGTGGCAAGTCCTTCG | qRT-PCR |
| CTnT-R | TGAATCTTCTGTGGCGAGTG | qRT-PCR |
| mMixl1-F | AGTTGCTGGAGCTCGTCTTC | qRT-PCR |
| mMixl1-R | AGGGCAATGGAGGAAAACTC | qRT-PCR |
| Nkx2-5-F | AAATCAATCACAGCCCCAAGTG | ChIP |
| Nkx2-5-R | GTTTATGGAAAACTCAAATAGCAG | ChIP |
| T-F | TCCCCTGCTCCTGTTACTGT | ChIP |
| T-R | GGCAGAGGGATGAGTCCAAAG | ChIP |
| Fgf3-F | CAGATCAGGCCCATCCCG | ChIP |
| Fgf3-R | GCGTGTGCTCCCAGCG | ChIP |
| PAX6-F | GTTTGAAAAGCGAACCGTGG | ChIP |
| PAX6-R | GATGGCTCAAGTGTGTTAATG | ChIP |
| HOXA10-F | GCGTCTTCTGGCCCATCAAT | ChIP |
| HOXA10-R | ACATGCTGAATACGATTAGCAA | ChIP |
| NEUROG1-F | TGTGCCACAACTTGGTGCTCA | ChIP |
| NEUROG1-F | CTCAGGGAGTGAATGGGCTT | ChIP |

**Table S2. Antibodies Used in this Study.**

| **Name** | **Brand** | **CAT number** | **Experiment** |
| --- | --- | --- | --- |
| mASXL2 | home made |  | western |
| mBAP1 | from HELIN |  | western |
| RYBP | millipore | AB3637 | ChIP |
| RING1B | CST | 5694s | ChIP |
| SUZ12 | CST | 3737S | ChIP |
| H2AK119ub1 | CST | 8240S | ChIP |
| H3K27me3 | CST | 9733s | ChIP |
| H3K4me3 | CST | 9751P | western |
| H3K4me1 | CST | 5326P | western |
| H3K27ac | Abcam | ab4729 | western |
| H3 | Active Motif | 61475 | western |
| α-actin | Abcam | ab5694 | IF |
| NANOG | Bethyl | A300-397A | IF |
| OCT4 | Abcam | ab184665 | IF |
